# Supplementary material for: Incidence and risk of periodontitis in obstructive sleep apnea: A meta-analysis
Source: PLoS One. 2022 Jul 21;17(7):e0271738. doi: 10.1371/journal.pone.0271738 (PMC9302852; doi:10.1371/journal.pone.0271738)
Supplement: S1 Checklist — (DOCX) [file pone.0271738.s001.docx]

| **Section and Topic** | **Item #** | **Checklist item** | **Location where item is reported** |
| --- | --- | --- | --- |
| **TITLE** | | |  |
| Title | 1 | Identify the report as a systematic review, meta-analysis, or a combination of the two. Page 1 |  |
| **ABSTRACT** | | |  |
| Abstract | 2 | See the PRISMA 2020 for Abstracts checklist. Page 2 |  |
| **INTRODUCTION** | | |  |
| Rationale | 3 | Describe the rationale for the review based on the context of existing knowledge.Page 3 and 4 |  |
| Objectives | 4 | Provide an explicit statement of the review's objective(s) or question(s).Page 4 |  |
| **METHODS** | | |  |
| Eligibility criteria | 5 | Specify the inclusion and exclusion criteria for the review.Page 5 |  |
| Search strategy | 6 | Provide a comprehensive search strategy for all databases, registries, and websites, including any filters and restrictions that may have been applied.Page 6 |  |
| Information sources | 7 | Specify all databases, registers, websites, organizations, and reference lists that were accessed or searched in order to conduct the study. Indicate the date of the most recent search or examination of each source.Page 6 |  |
| Selection process | 8 | Describe the technique used to evaluate if a study fulfilled the review's inclusion criteria, including the number of reviewers who reviewed each record and report retrieved, their independence, and, if appropriate, details about the automated technologies utilized in the process.Page 5 and 6 |  |
| Data collection process | 9 | Describe the method for extracting data from the report and any process for obtaining and validating data from the investigator.Page 5 and S1 Table |  |
| Data items | 10 | Indicate how many reviewers reviewed each record and each report retrieved, whether they worked independently, and, if appropriate, information of the automated technologies utilized.Page 5 and 6 |  |
|  |  |  |  |
| Study risk of bias assessment | 11 | Specify the methods used to assess the risk of bias in the included studies, including details of the tools used, how many reviewers assessed each study, whether they worked independently, and if applicable, details of the automated tools used in the process.Page 6 and 7 |  |
| Effect measures | 12 | For each outcome, specify the effect measure (e.g., risk ratio, mean difference) to be used in the synthesis or presentation of the results.Page 7 |  |
| Synthesis methods | 13a | Describe the process used to determine which studies qualify for each composite study (e.g., tabulating the intervention characteristics of the studies and comparing them to the planned group for each composite study (item #5)) .Page 6 and 7 |  |
|  | 13b | Describe any methods needed to prepare data for presentation or synthesis, such as processing missing summary statistics, or data transformation.Page 7 |  |
|  | 13c | Describe any methods used to tabulate or visually display results of individual studies and syntheses.Page 6 and 7 |  |
|  | 13d | Describe any methods used to synthesize the results and provide a rationale for the selection. If a meta-analysis was performed, describe the model, the method used to determine the presence and extent of statistical heterogeneity, and the software package used.Page 6 and 7 |  |
|  | 13e | Describe any methods used to explore possible causes of heterogeneity among study results.Page 6 and 7 |  |
|  | 13f | Describe any sensitivity analysis performed to assess the robustness of the combined results.Page 6 and 7 |  |
| Reporting bias assessment | 14 | Describe any methods used to assess risk of bias due to missing results in a synthesis (arising from reporting biases).Page 6 and 7 |  |
| Certainty assessment | 15 | Describe any methods used to assess the certainty (or credibility) of the body of evidence for the results.Page 6 and 7 |  |
| **RESULTS** | | |  |
| Study selection | 16a | Describe the results of the search and selection process, from the number of records found in the search to the number of studies included in the review.Page 8 and Figure 1 |  |
|  | 16b | Cite studies that might appear to meet the inclusion criteria, but which were excluded, and explain why they were excluded.Page 8 and Figure 1 |  |
| Study characteristics | 17 | Cite each included study and present its characteristics.Page 8 and Table 1 |  |
| Risk of bias in studies | 18 | Present assessments of risk of bias for each included study.Page 8 and Table 2 |  |
| Results of individual studies | 19 | For all outcomes, present, for each study: (a) summary statistics for each group (where appropriate) and (b) an effect estimate and its precision (e.g. confidence/credible interval), ideally using structured tables or plots.Page 9 and Table 1 |  |
| Results of syntheses | 20a | For each synthesis, briefly summarise the characteristics and risk of bias among contributing studies.Page 9 and Table 2 |  |
|  | 20b | Present results of all statistical syntheses conducted. If meta-analysis was done, present for each the summary estimate and its precision (e.g. confidence/credible interval) and measures of statistical heterogeneity. If comparing groups, describe the direction of the effect.Page 10 and Figure 2, 3 and 5 |  |
|  | 20c | Present results of all investigations of possible causes of heterogeneity among study results.Page 11 and Figure 4 and 6 |  |
|  | 20d | Present results of all sensitivity analyses conducted to assess the robustness of the synthesized results.Page 10 and 11 |  |
| Reporting biases | 21 | Present assessments of risk of bias due to missing results (arising from reporting biases) for each synthesis assessed.Page 10 and 11 |  |
| Certainty of evidence | 22 | Present assessments of certainty (or confidence) in the body of evidence for each outcome assessed.Page 10 and 11 |  |
| **DISCUSSION** | | |  |
| Discussion | 23a | Provide a general interpretation of the results in the context of other evidence. Page 12 and 13 |  |
|  | 23b | Discuss any limitations of the evidence included in the review. Page 13 and 14 |  |
|  | 23c | Discuss any limitations of the review processes used.Page 14 and 15 |  |
|  | 23d | Discuss implications of the results for practice, policy, and future research.Page 15 |  |
| **OTHER INFORMATION** | | |  |
| Registration and protocol | 24a | Provide registration information for the review, including register name and registration number, or state that the review was not registered.Page 4 and 5 |  |
|  | 24b | Indicate where the review protocol can be accessed, or state that a protocol was not prepared.Page 4 and 5 |  |
|  | 24c | Describe and explain any amendments to information provided at registration or in the protocol.Page 4 and 5 |  |
| Support | 25 | Describe sources of financial or non-financial support for the review, and the role of the funders or sponsors in the review.Page16 |  |
| Competing interests | 26 | Declare any competing interests of review authors..Page16 |  |
| Availability of data, code and other materials | 27 | Report which of the following are publicly available and where they can be found: template data collection forms; data extracted from included studies; data used for all analyses; analytic code; any other materials used in the review.Page16 and 17 |  |

*From:*  Page MJ, McKenzie JE, Bossuyt PM, Boutron I, Hoffmann TC, Mulrow CD, et al. The PRISMA 2020 statement: an updated guideline for reporting systematic reviews. BMJ 2021;372:n71. doi: 10.1136/bmj.n71

For more information, visit: <http://www.prisma-statement.org/>
