# Supplementary material for: Incidence and risk of periodontitis in obstructive sleep apnea: A meta-analysis
Source: PLoS One. 2022 Jul 21;17(7):e0271738. doi: 10.1371/journal.pone.0271738 (PMC9302852; doi:10.1371/journal.pone.0271738)
Supplement: S1 Table — (DOCX) [file pone.0271738.s002.docx]

S1 Table. Literature Retrieval Strategy.

| **PubMed** | | |
| --- | --- | --- |
| #1 | Search "Sleep Apnea, Obstructive"[Mesh] | 24627 |
| #2 | Search ((((Sleep-related breathing disorders[Title/Abstract]) OR (sleep disordered breathing[Title/Abstract])) OR (Obstructive Sleep Apnea*[Title/Abstract])) OR (Obstructive Sleep Apnea Syndrom[Title/Abstract])) OR (OSAHS[Title/Abstract]) | 33210 |
| #3 | #1 OR #2 | 39859 |
| #4 | Search "Periodontitis"[Mesh] | 32980 |
| #5 | Search ((periodontal[Title/Abstract]) OR (gum disease[Title/Abstract])) OR (periodontal disease[Title/Abstract]) | 65788 |
| #6 | Search ((((((((Loose teeth[Title/Abstract]) OR (attachment loss[Title/Abstract])) OR (alveolar bone loss[Title/Abstract])) OR (clinical attachment loss[Title/Abstract])) OR (Clinical attachment level[Title/Abstract])) OR (pocket depth[Title/Abstract])) OR (tooth loss[Title/Abstract])) OR (Dental plaque[Title/Abstract])) OR (oral hygiene[Title/Abstract]) | 34616 |
| #7 | #4 OR #5 OR #6 | 99814 |
| #8 | #3 AND #7 | 74 |
| **Embase** | | |
| #1 | 'sleep apnea, obstructive'/exp | 90957 |
| #2 | 'Sleep-related breathing disorders':ab,ti OR 'sleep disordered breathing':ab,ti OR 'Obstructive Sleep Apnea*':ab,ti OR 'Obstructive Sleep Apnea Syndrome':ab,ti OR 'OSAHS':ab,ti | 63031 |
| #3 | #1 OR #2 | 94453 |
| #4 | 'periodontitis'/exp | 50012 |
| #5 | 'periodontal disease':ab,ti OR 'gum disease':ab,ti OR 'periodontal':ab,ti OR 'attachment loss':ab,ti OR 'alveolar bone loss':ab,ti OR 'clinical attachment loss':ab,ti OR 'Clinical attachment level':ab,ti OR 'pocket depth':ab,ti OR 'Dental plaque':ab,ti OR 'oral hygiene':ab,ti OR 'Loose teeth':ab,ti OR 'tooth loss':ab,ti | 88368 |
| #6 | #4 OR #5 | 110911 |
| #7 | #3 AND #6 | 169 |
|  | **Web of since** |  |
| #1 | Search TS=(Sleep-related breathing disorders OR sleep disordered breathing OR Sleep Apnea, Obstructive OR Obstructive Sleep Apnea* OR Obstructive Sleep Apnea Syndrome OR OSAHS) | 64260 |
| #2 | Search TS=(Loose teeth OR Periodontitis OR periodontal disease OR gum disease OR periodontal OR attachment loss OR alveolar bone loss OR clinical attachment loss OR Clinical attachment level OR pocket depth OR tooth loss OR Dental plaque OR oral hygiene) | 224257 |
| #4 | #1 AND #2 | 229 |
